# Whole genome sequencing of *Moraxella bovoculi* reveals high genetic diversity and evidence for interspecies recombination at multiple loci

**DOI:** 10.1371/journal.pone.0209113

**Published:** 2018-12-17

**Authors:** Aaron M. Dickey, Gennie Schuller, J. Dustin Loy, Michael L. Clawson

**Affiliations:** 1 Genetics, Breeding, and Animal Health Research Unit, U.S. Meat Animal Research Center, Agricultural Research Service, United States Department of Agriculture, Clay Center, Nebraska, United States of America; 2 Veterinary Diagnostic Center, School of Veterinary Medicine and Biomedical Sciences, University of Nebraska-Lincoln, Lincoln, Nebraska, United States of America; Erasmus Medical Center, NETHERLANDS

## Abstract

*Moraxella bovoculi* is frequently cultured from the ocular secretions and conjunctiva of cattle with Infectious Bovine Keratoconjunctivitis (IBK). Previous work has shown that single nucleotide polymorphism (SNP) diversity in this species is quite high with 81,284 SNPs identified in eight genomes representing two distinct genotypes isolated from IBK affected eyes (genotype 1) and the nasopharynx of cattle without clinical IBK signs (genotype 2), respectively. The goals of this study were to identify SNPs from a collection of geographically diverse and epidemiologically unlinked *M*. *bovoculi* strains from the eyes of IBK positive cattle (n = 183) and another from the eyes of cattle (most from a single population at a single time-point) without signs of IBK (n = 63) and to characterize the genetic diversity. Strains of both genotypes were identified from the eyes of cattle without IBK signs. Only genotype 1 strains were identified from IBK affected eyes, however, these strains were isolated before the discovery of genotype 2, and the protocol for their isolation would have preferentially selected genotype 1 *M*. *bovoculi*. The core genome comprised ~74% of the whole and contained >127,000 filtered SNPs. More than 80% of these characterize diversity within genotype 1 while 23,611 SNPs (~18%) delimit the two major genotypes. Genotype 2 strains lacked a repeats-in-toxin (RTX) putative pathogenesis factor and any of ten putative antibiotic resistance genes carried within a genomic island. Within genotype 1, prevalence of these elements was 0.85 and 0.12 respectively in strains from eyes that were IBK positive. Recombination appears to be an important source of genetic diversity for genotype 1 and undermines the utility of ribosomal-locus-based species identification. The extremely high genetic diversity in genotype 1 presents a challenge to the development of an efficacious vaccine directed against them, however, several low-diversity pilin-like genes were identified. Finally, the genotype-defining SNPs described in this study are a resource that can facilitate the development of more accurate *M*. *bovoculi* diagnostic tests.

## Introduction

Infectious Bovine Keratoconjunctivitis (IBK) is an important disease that affects 2–10% of beef cattle in the United States [1–3] and is associated with pain [3–5], blindness in severe cases [4], and reduced weight gain [6–7]. In addition to animal health and welfare concerns, the economic impact of IBK can be profound with estimates exceeding 150 million in the US alone in direct and indirect economic losses [8].

The only experimentally reproducible etiologic agent for IBK is *Moraxella bovis* [3,9–10], though other organisms have been detected in high frequencies during outbreaks, including *Mycoplasma* spp. [11] and *Moraxella bovoculi* [12]. *M*. *bovis* is transmitted by direct contact [13], ocular discharge [14], and mechanical vectors; principally, the face fly [2,15–17]. Thus, IBK prevention can be assisted via fly management [16]. Eye patches and shade of quarantine animals can reduce fly contact and ultraviolet exposure, which can predispose animals to disease and aggravate disease severity [18]. IBK can be successfully treated, and two drugs are approved in the US for treatment (tulathromycin and oxytetracycline) with strains showing broad susceptibility [19]. The efficacy of currently available licensed and autogenously produced vaccines is controversial, however, homologous bacterins have shown to reduce disease incidence and severity [2–3,6,20–24].

The gram-negative coccobacillus, *M*. *bovoculi*, has been widely associated with IBK in the absence of *M*. *bovis* since its initial description in 2007 [4,12,25–30]. *M*. *bovoculi* is the most frequently isolated bacteria from clinical cases [12] and its presence correlates with IBK lesions [29]. While this bacterium is generally associated with cattle eyes, it has also been found in other mammalian hosts [31–32] and niches [33–35]. While the type strain has not been shown to induce ocular lesions experimentally [3], *M*. *bovoculi* may exacerbate *M*. *bovis* infections or play other roles in the IBK disease state.

Previously, >81K SNPs were identified from 8 complete *M*. *bovoculi* genomes indicative of very high genetic diversity in this species [35]. Furthermore, distinct major genotypes, with differing assortments of virulence factors, were found inhabiting the eyes of IBK positive cattle and the nasopharynx of non-IBK cattle (genotypes 1 and 2 respectively), suggesting that some strains may vary in their propensity to associate with IBK. The aim of this study was to more fully characterize *M*. *bovoculi* genetic diversity found in IBK and apparently healthy cattle eyes.

## Materials and methods

A total of 246 *Moraxella bovoculi* strains were used in this study. Of these, 183 were epidemiologically unlinked clinical strains originating from IBK cattle in 22 US States with heavy bias toward the state of Nebraska (60.1% of strains) and bordering states (10.9%) (Table 1, S1 Table). These strains were recovered from eye swabs sent to the University of Nebraska-Lincoln Veterinary Diagnostic Center by submitting veterinarians for diagnostic assessment of IBK cases [12]. Importantly, the 183 strains were assembled into a collection prior to the discovery of genotype 2 *M*. *bovoculi*, and were identified in part using colony morphologies, gram staining, oxidase activity and a PCR-RFLP technique that would have misidentified genotype 2 [35–36]. The other 63 strains were obtained by swabbing the eyes of deceased and living animals that did not have signs of IBK (S1 Table). All but six of the 63 strains were obtained from 57 cattle at the US Meat Animal Research Center in the fall of 2015 and were identified through culture, PCR, ribosomal DNA (rDNA) analyses, and MALDI-TOF procedures that would detect either genotype 1 or 2 *M*. *bovoculi* [37]. Use of cattle in the latter group were collectively approved by the Institutional Animal Care and Use Committees of the University of Nebraska-Lincoln (IACUC Project #1291) and the US Meat Animal Research Center (2017-10-11 EO #24.5).

**Table 1:** Summary of strains included in this study.

Strain count	IBK Signs	Collection location	Identified after the discovery of genotype 2	Prevalence of full or partial antibiotic resistance element in genotype 1	Prevalence of repeats-in-toxin in genotype 1	Genotype
110	+	Nebraska, USA	No	7.27%	88.18%	1
73	+	21 other US States	No	19.18%	79.45%	1
6	-	University of Nebraska	No	0%	33.33%	1
57	-	US Meat Animal Research Center	Yes	0%	58.06%	1 and 2

To construct DNA libraries for sequencing, cultures were streaked from frozen stocks on chocolate agar plates and passaged twice overnight at 37C° and 5% CO_2_. Single colonies were then inoculated in brain heart infusion broth and grown overnight without shaking. DNA was extracted from the cultures using an UltraClean -htp 96 Well Microbial DNA Isolation kit (Mo Bio, Carlsbad, CA) and libraries were developed with a Nextera XT kit (Illumina, San Diego, CA) and AMpure beads (Pacific Biosystems, Menlo Park, CA). Whole genome sequencing was then conducted on a MiSeq machine (Illumina) to generate 300 bp paired reads.

Reads from each library were mapped to a complete *M*. *bovoculi* reference genome [34] (GenBank CP011374) using Geneious v8 software (Biomatters, Aukland, NZ). The libraries were quality control checked in Geneious to ensure they had >10X coverage to the reference genome and consisted of a single clonal strain with a single allele at each reference genome position. The consensus rDNA sequence of each mapped strain was extracted from the raw reads, and a maximum likelihood (ML) tree of the sequences was constructed via a custom in-house bioinformatic pipeline to ensure all strains were *M*. *bovoculi* [37]. The pipeline utilized the Burrows-Wheeler alignment (bwa) [38], samtools and bcftools [39], and Clustal Omega [40] for mapping, SNP calling, and consensus sequence alignment respectively. DnaSP [41], PartitionFinder [42], and RAxML [43] were then used for indel extraction, model optimization, and tree construction respectively. The ML tree of the rDNA sequences can be found in reference 37. Single nucleotide polymorphisms (SNPs) were identified and extracted from consensus of each mapped strain genome using PARSNP [44]. Within PARSNP, default filters based on alignment uncertainty and proximity to a localized collinear block boundary were enforced but SNPs with an ambiguous base call in one-or-more genomes were retained if they were ambiguous in <5% of genomes. SNPs from the repeated portions of the core genome, such as ribosomal RNA (rRNA) genes, *tuf* and surrounding regions of the genome, were also removed from the core genome SNP dataset. PARSNP utilized FastTree 2 [45] for approximate Maximum Likelihood (aML) phylogenetic inference. Initial trees were star-like with many long branch lengths and multiple nodes with low support so PARSNP was run a second time with the recombination detection filter enabled.

Since it was previously reported that not all IBK associated *M*. *bovoculi* strains contain any of ten putative antibiotic resistance (AR) genes located within a genomic island, or a repeats-in-toxin (RTX) putative pathogenesis factor [35], strains were characterized based on the presence/absence of these genomic elements. PARSNP was then run a third time only on those *M*. *bovoculi* genomes containing the RTX operon, so that RTX SNPs could be extracted for detailed analysis.

The SNP alignment (S1 Dataset) for 220 genotype 1 strains, minus SNPs identified as recombinant with PARSNP, was used to perform a recombination test with PhiPack [46]. The alignment was also used to construct a phylogenetic network, which accounts for recombination, in Splitstree [47]. In addition, suspected interspecies mosaics at rRNA genes and the RTX operon were readily visualized in Geneious as dense clusters of polymorphisms divergent from the consensus sequence. To confirm interspecies mosaicism, potential recombination events were queried against the GenBank Nucleotide database using BLAST [48].

A single pilin gene had been previously identified in the *M*. *bovoculi* draft genome, AOMT00000000 contig 37 [49] and annotated PilA. This gene was annotated as a prepilin-type N-terminal cleavage/methylation domain-containing protein CDS in CP011374 but was determined to be the ortholog to this pilin gene based on 99.3% protein identity. Two additional pilin-like genes had also each been annotated as a prepilin-type N-terminal cleavage/methylation domain-containing protein CDS in CP011374 [35]. These additional genes are contained within a single operon immediately adjacent to one another in CP011374 (coordinates 503488–505002). SNPs from both genes were identified by PARSNP indicating they are also part of the core *M*. *bovoculi* genome. Because of the importance of pilin serotype in *M*. *bovis* and the new GenBank pilin annotations not previously discussed in the literature, a hidden Markov model (HMM) of the type IV pre-pilin domain (TIGR02532) was built to search for any additional pilin-like genes in the reference genome.

To conduct the HMM search, SNPs were first identified in the PilA gene, using the mapping, SNP calling, and alignment portion of the custom bioinformatic pipeline described above and in reference 37. Though this gene is in the core genome of *M*. *bovoculi*, PARSNP did not recover any SNPs at this locus. Second, 13 unique TIGR02532 amino acid haplotypes were identified from the two pilin-like genes located at coordinates 503488–505002 in CP011374. Third, iterative PSI-BLAST [50] searches were conducted via the National Center for Biotechnology Information (NCBI) webserver on June 28, 2018 using default settings on the consensus of these haplotypes. The PSI-BLAST searches were first conducted against *M*. *ovis* and *M*. *bovis* and progressively expanded to *Moraxella* sp. and members of the family *Moraxellaceae* using the consensus from the previous progression in each successive search. Multiple PSI-BLAST iterations were conducted at each taxonomic level until no additional haplotypes were identified at the default P-value threshold. Only 2 iterations were conducted for the search against members of the *Moraxellaceae* since the webserver returned out-of-memory errors after 702 sequences were found. One hundred of the most diverse TIGR02532 members and 14 of the top listed sequences as identified by NCBI were included in the HMM for a total of 896 haplotypes. The HMM was built and all annotated protein coding sequences of CP011374 were queried using HMMER 3.1b2 [51].

## Results

Mean coverage for the 246 sequenced genomes ranged from 15.0 to 148.3 (median: 47.6; interquartile range: 30.3). The GC content ranged from 45.5–46.3%. The core genome comprised ~74% of reference genome GenBank CP011374. A total of 142992 SNPs within the core genome were identified by PARSNP and, of those, 127918 met our filter criteria.

**Fig 1:**
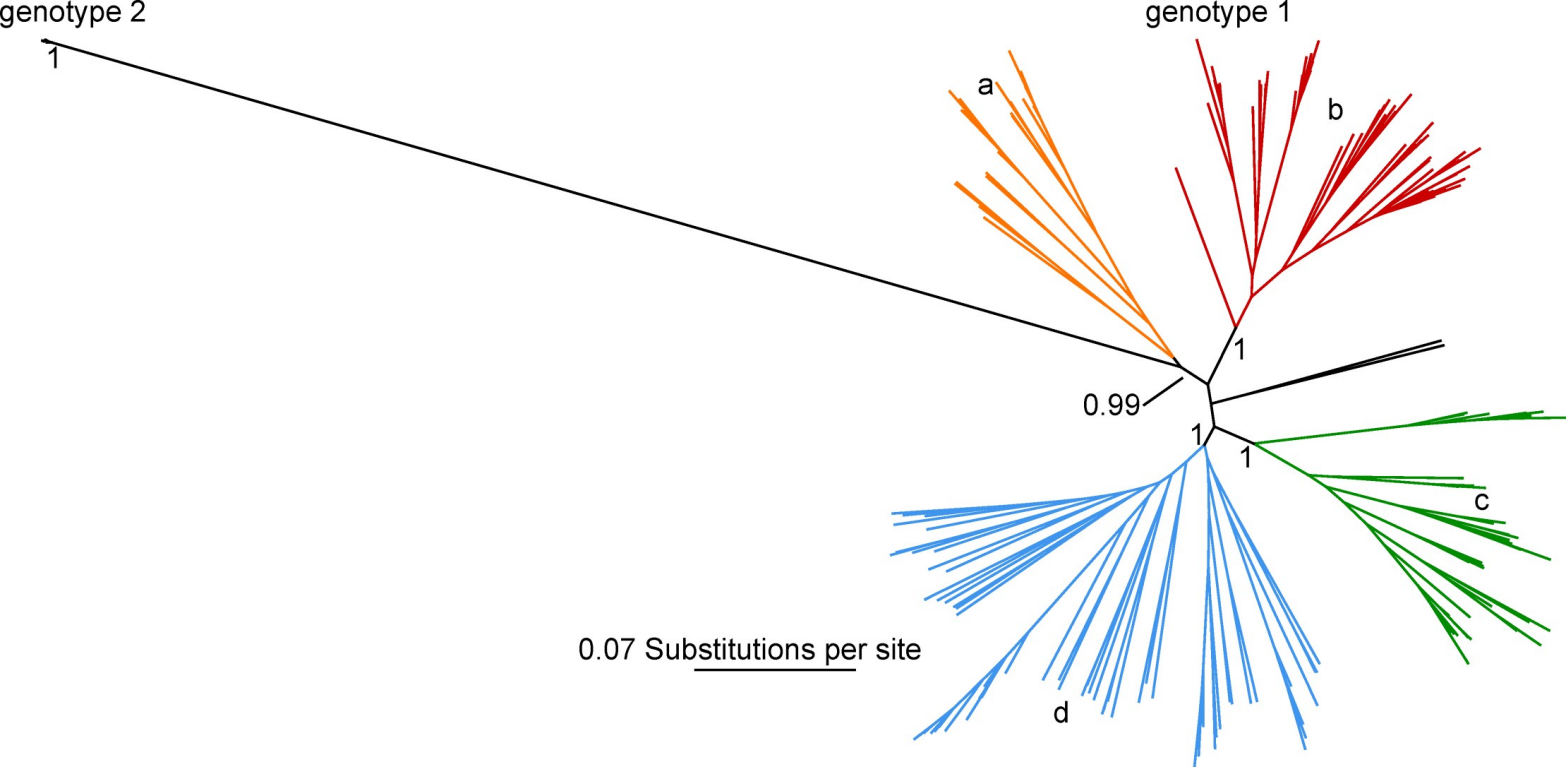
Approximate maximum likelihood unrooted tree of 246 *M*. *bovoculi* whole genome single nucleotide polymorphisms (SNP) profiles with Phi Pack identified recombination SNPs removed to increase node support within genotype 1. Four well supported subtypes of genotype 1(a-d) are color coded.

An aML phylogeny was constructed with 4014 SNPs removed that were identified by PARSNP as recombinant (Fig 1). The tree highlights the large divergence between 26 low-diversity genotype 2 genomes and 220 high-diversity genotype 1 genomes. The 26 genotype 2 strains were all collected from non-IBK eyes. A total of 23611 SNPs (S2 Table) diagnostically separate the two major genotypes, that is each allele at these sites is exclusively found in one of the two clades. The tree in Fig 1 also shows four subtypes within genotype 1.

**Fig 2:**
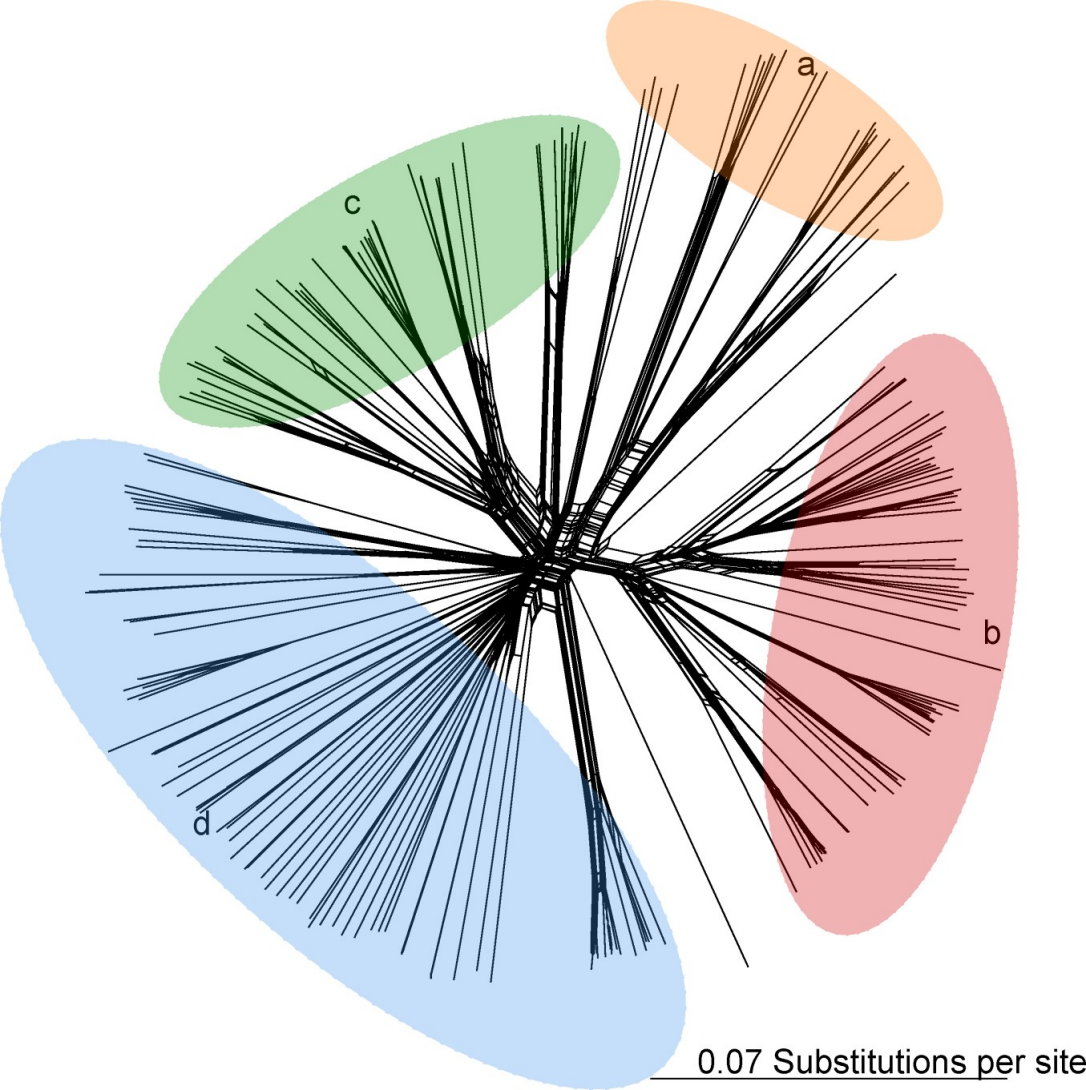
Neighbor-net tree of single nucleotide polymorphism genotypes from 220 *M*. *bovoculi* genotype 1 strains. Letters, ellipsoids and colors denote subtypes from Fig 1.

Despite the removal of 4014 recombinant SNP alleles, a PHI test for recombination on the remaining SNP dataset was positive (observed Φ_*w*_ = 0.38, expected Φ_*w*_ = 0.57, p = 0.00). Thus, the relationships among genotype 1 were also examined by a neighbor-net tree, which could account for recombination (Fig 2). The four diverse subtypes were retained in the neighbor net tree, however, due to high recombination, no diagnostic SNPs were identified that tag any of the subtypes, even though these groups have high phylogenetic support in the aML tree.

Neither RTX or any of the 10 previously reported AR genes were found in genotype 2 strains (S1 Table). The frequency of the RTX operon in genotype 1 isolated from IBK animals was 85% vs 54% in genotype 1 from non-IBK animals (Table 1, S1 Table). The frequency of any of the 10 putative AR genes in genotype 1 *M*. *bovoculi* isolated from IBK animals was 12% vs 0% in genotype 1 from non-IBK animals (Table 1). Twenty-two genotype 1 strains had between 2 and 10 of the genes in the island (S1 Table). The most common of these were tetracycline resistance genes, which were found in all 22 strains. The least common were genes 6 & 7, *msr(E)* conferring resistance to macrolide, lincosamide and streptograminB, and *mph(E)* conferring macrolide resistance, which were found in 9 of the 22 strains. Regarding the subtypes of genotype 1, the RTX frequency was 100%, 91%, 92%, and 59% in subtypes a-d respectively and AR frequency was 9%, 20%, 6%, and 5% in subtypes a-d respectively.

**Fig 3:**
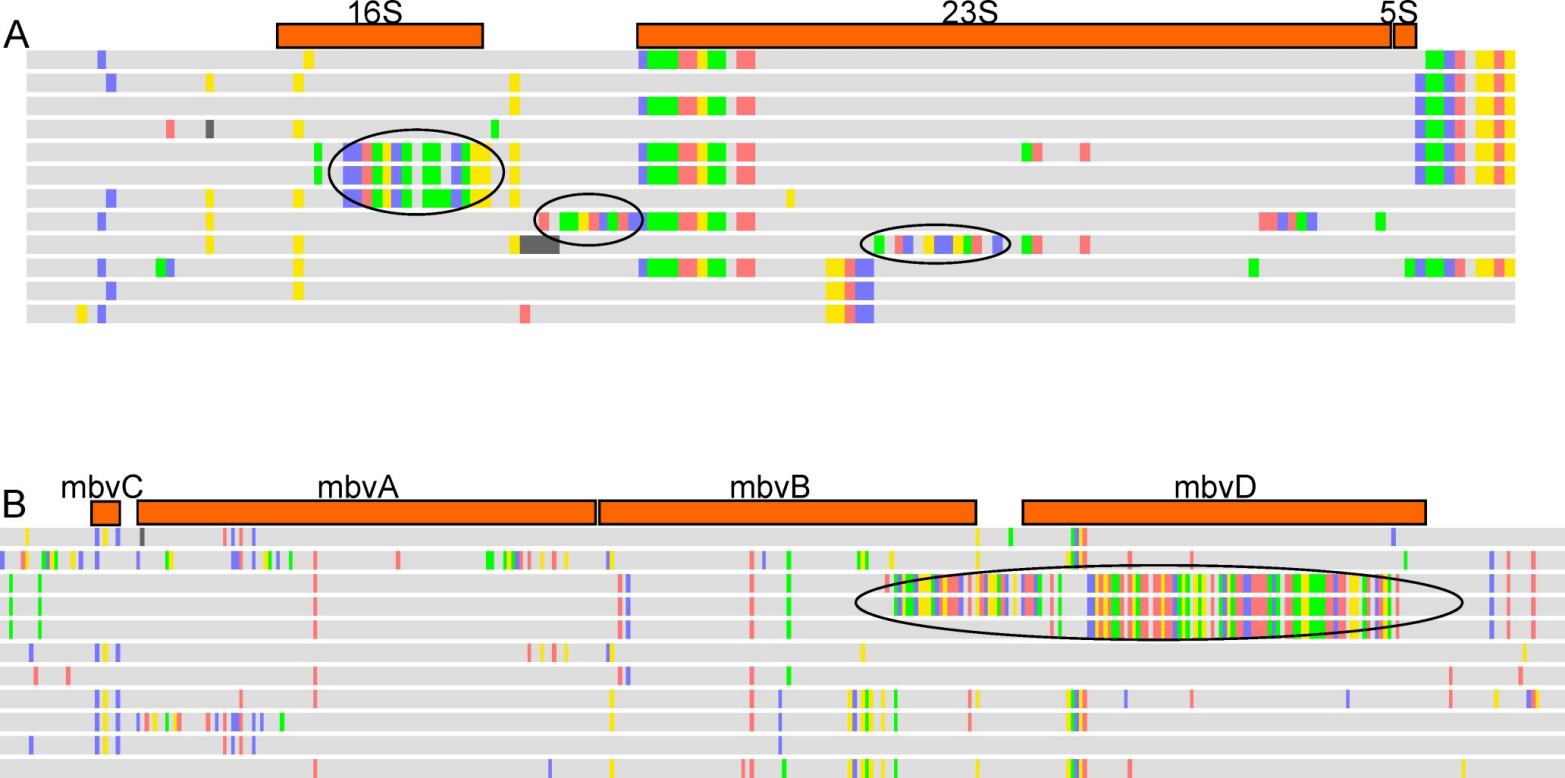
**Partial *Moraxella bovoculi* single nucleotide polymorphism (SNP) alignments of A) a rRNA locus and B) the RTX locus with constituent genes appearing as orange bars above alignments.** Multi-colored SNP blocks (circled) are highly divergent from the grey consensus sequence and hypothesized to be interspecies mosaics. Confirmed recombinants via BLAST are listed in Table 2.

Apparent recombination was detected at both the rRNA and RTX operon loci. The rRNA genes and surrounding non-coding repetitive regions contained 686 SNPs that did not meet our filter criteria for SNP identification. Visualizing the SNP profiles in Geneious showed dense SNP clusters in some strains (Fig 3), which may reflect recombination with *M*. *bovis*, or a species closely related to *M*. *bovis*, as the hypothesized recombinants placed phylogenetically intermediate between the two species [37]. The RTX operon contained an additional 338 SNPs that did not meet our filter criteria for SNP identification, and several strains also contained dense SNP clusters indicative of potential recombination at the locus (Fig 3). Several of these putative interspecies recombination events were confirmed using BLAST (Table 2). Ten strains are *M*. *bovis* mosaics at RTX operon genes *mbvB* and *mbvD*, three are mosaics at *mbvD* only, and six strains are interspecies mosaics at rRNA genes. A single strain, Mb68506, is recombinant at both loci.

**Table 2:** Interspecies recombinant sequences at the repeats-in-toxin and ribosomal RNA loci.

Strain	Query^a^	Top BLAST Hit; Range	Hit Species	Hit Strain	Coverage	Identity	Evalue	Gene(s)
Mb68490	559504–560901	AF205359.3; 6545–7943	*Moraxella bovis*	Tifton 1	100.0%	100.0%	0	RTX-*mbvB*/*mbvD*
Mb68487	559504–560901	AF205359.3; 6545–7943	*Moraxella bovis*	Tifton 1	100.0%	100.0%	0	RTX-*mbvB*/*mbvD*
Mb57897	560086–561351	AF205359.3; 7145–8393	*Moraxella bovis*	Tifton 1	100.0%	100.0%	0	RTX-*mbvB*/*mbvD*
Mb58051	560086–561351	AF205359.3; 7145–8393	*Moraxella bovis*	Tifton 1	100.0%	100.0%	0	RTX-*mbvB*/*mbvD*
Mb68506	560086–561351	AF205359.3; 7145–8393	*Moraxella bovis*	Tifton 1	100.0%	100.0%	0	RTX-*mbvB*/*mbvD*
Mb68527	560086–561351	AF205359.3; 7145–8393	*Moraxella bovis*	Tifton 1	100.0%	100.0%	0	RTX-*mbvB*/*mbvD*
Mb68531	560086–561351	AF205359.3; 7145–8393	*Moraxella bovis*	Tifton 1	100.0%	100.0%	0	RTX-*mbvB*/*mbvD*
Mb68532	560086–561351	AF205359.3; 7145–8393	*Moraxella bovis*	Tifton 1	100.0%	100.0%	0	RTX-*mbvB*/*mbvD*
Mb68536	560086–561351	AF205359.3; 7145–8393	*Moraxella bovis*	Tifton 1	100.0%	100.0%	0	RTX-*mbvB*/*mbvD*
Mb58057	560567–561351	AF205359.3; 7609–8393	*Moraxella bovis*	Tifton 1	100.0%	100.0%	0	RTX-*mbvD*
Mb68490	560960–561293	AF205359.3; 8002–8335	*Moraxella bovis*	Tifton 1	100.0%	100.0%	5E-173	RTX-*mbvD*
Mb68487	560960–561293	AF205359.3; 8002–8335	*Moraxella bovis*	Tifton 1	100.0%	100.0%	5E-173	RTX-*mbvD*
Mb68506	22003–22303	JN001958.1; 89–389	*Moraxella bovis*	18 named strains	100.0%	100.0%	1E-154	rRNA-16S
Mb57872	22858–22900	LT838786.1; 908–950	*M*. *bovis*, *M*. *pluranimalium*, *M*. *sp*.	11 named *M*. *bovis* strains	100.0%	100.0%	1E-12	rRNA-16S(V6)
Mb58083	22858–22900	LT838786.1; 908–950	*M*. *bovis*, *M*. *pluranimalium*, *M*. *sp*.	11 named *M*. *bovis* strains	100.0%	100.0%	1E-12	rRNA-16S(V6)
Mb58062	22858–22900	LT838786.1; 908–950	*M*. *bovis*, *M*. *pluranimalium*, *M*. *sp*.	11 named *M*. *bovis* strains	100.0%	97.7%	6E-11	rRNA-16S(V6)
Mb58056	23834–23865	CP011158.1; 23911–23947	*Moraxella ovis*	3 named strains	100.0%	100.0%	2E-09	rRNA-intergenic spacer
Mb58073	24706–25325	DQ647927.1; 3196–3815	*Moraxella bovis*	Tifton 1	100.0%	99.7%	0	rRNA-23S

^a^Positions in reference genome CP011374

In addition to the three prepilin-type N-terminal cleavage/methylation domain-containing protein coding genes annotated in the reference *M*. *bovoculi* genome CP011374, a fourth gene that is likely either a type II pilin or pseudopilin was identified in CP011374 (TIGR02532 HMM E-Value 0.0029), (Table 3). This gene was annotated as a hypothetical gene in CP011374. Within the type IV pre-pilin TIGR02532 domain GFxxxE this gene encodes the amino acid sequence “GFTFIQ” and was predicted via blastp to contain the pfam12019(*GspH*) conserved domain (Table 3). Of the four pilins or pseudopillin genes listed in Table 3, the one with a specific BLAST hit to the Pilin domain (pfam0014), and a non-specific hit to the PilA conserved domain (COG4969), was the most highly divergent between *M*. *bovoculi* genotype 1 and genotype 2 with >28% of its reference length comprised of diagnostic SNPs. This is the same gene that is orthologous to the PilA gene in AOMT00000000. The remaining genes showed low divergence (~1%) between the two genotypes (Table 3).

**Table 3:** Characteristics of four pilin-like genes in *M*. *bovoculi*.

Gene^a^	TIGR02532 (IV_pilin_ GFxxxE) domain	Specific blastp conserved domain hit	Non-specific blastp conserved domain hits	Superfamilies	Reference coordinates	Length	Diagnostic SNPs separating genotype 1 and 2	Total SNPs
AAX09_ RS01900	positive	Pilin	PilA	Pilin, N_methyl	394367–394825	459	130	164
AAX09_ RS02410	positive	N-terminal methylation motif	PilV, type IV pilV, PRK10557	PilV, N_methyl, PRK10557	503488–504012	525	3	46
AAX09_ RS02415	positive	N-terminal methylation motif	PilE, PRK100557	PilW, PRK10557, N-terminal methylation motif	504013–505002	990	12	75
AAX09_ RS07765	negative	GspH	none	Gsph	1677589–1677056	534	3	40

^a^locus tag in NZ_CP011374

## Discussion

This study developed a SNP-based classification system for *M*. *bovoculi* from 183 IBK cattle case submissions covering a large portion of the United States and 63 non-IBK eye strains. This system differentiates two major genotypes of *M*. *bovoculi*. Strains belonging to genotype 1 have incredibly high SNP diversity, some of which we have attributed to interspecies recombination at both the 16S and RTX loci. Lastly, the frequencies of pathogenic and AR genes in Genotype 1 were higher in strains from IBK versus non-IBK eyes.

While genotype 2 was originally characterized based on strains recovered from the deep nasopharynx of cattle [35], genotype 2 strains are not limited to that niche. Neither are genotype 1 strains solely found in IBK eyes. Strains of both major genotypes can be isolated and grown from the eyes of apparently healthy cattle. Since genotype 2 was only recently characterized, and our collection of strains from IBK eyes were assembled with a protocol that would have excluded genotype 2 strains that may also have been present in the affected eyes, the frequency and prevalence of genotype 2 *M*. *bovoculi* in IBK eyes is unknown [35–36]. While genotype 2 does contain multiple pilin genes, all strains examined so far lack the RTX operon [35], so it may not associate with IBK as frequently as genotype 1 due to a lack of pathogenic determinants including the RTX operon. The low SNP diversity seen in the 26 genotype 2 genomes is potentially impacted by very low spatio-temporal sampling, however low SNP diversity was also seen within this genotype collected from multiple states in our previous study [35].

Of the 127918 SNPs identified with PARSNP, ~18% are diagnostic for separating the two major genotypes. This is a significant decrease from [35] in both the number (~23K vs ~40K) and percentage (~18 vs ~50) of diagnostic SNPs differentiating these clades. There are several causes for this change. Methodologically, SNPs were identified in [35] using MAUVE [52], which uses a more precise but substantially slower alignment algorithm [44]. In contrast, PARSNP was a much more feasible option for this study containing 30-fold more genomes, which were mapped to a reference rather than circularized and completed. Thus, many SNPs have been filtered in this study due to alignment uncertainty, and this uncertainty is predicted to be greater for high divergence orthologs among the two genotypes. For example, in [35], MAUVE identified 128 of 144 genotype-differentiating SNPs from the *M*. *bovoculi* PilA gene, while in the present study no SNPs were identified by PARSNP even though the PilA gene is present in all genomes. While this change is predicted to reduce the number of SNPs found, the large increase in genome count should increase the SNP count. Indeed, there is a >2-fold increase in the number of SNPs variable within genotype 1. Due to these two factors, the type-defining SNPs identified in this study can be viewed as a high-quality conservative subset of the total, which come from more conserved portions of the *M*. *bovoculi* core genome.

While no SNPs from the PilA gene were recovered by PARSNP, 164 SNPs from this locus were obtained using a custom in-house pipeline of which 130 diagnostically separated genotypes 1 and 2. This represents a >23-fold higher divergence among genotypes than seen in the other three pilin-like genes. The orthology of the PilA gene in *M*. *bovoculi* and the oft-studied homolog of *M*. *bovis* [53–60] is not straightforward, mainly because the *M*. *bovis* PilA gene undergoes inversion mediated phase variation that renders I and Q versions of the gene. We looked for evidence of an I/Q phase shift in our strains of *M*. *bovoculi* and found none. Additionally, the PilA genes of *M*. *bovis* and *M*. *bovoculi* are ≥60% divergent at the amino acid level [49]. Extreme diversifying selection at this locus may be partially responsible for widespread challenges in development of an effective IBK vaccine [20–23], several of which are pilin based [24, 57–60]. The use of HMM approaches may predict additional pilin-like genes in *M*. *bovis* as it has for *M*. *bovoculi*, and these may be critical to further understanding host-pathogen interaction.

Given that almost all non-IBK *M*. *bovoculi* strains were collected at the US Meat Animal Research Center (USMARC) in the fall of 2015 versus the epidemiologically unlinked collection of IBK positive eye strains, there were elemental frequency differences among genotype 1 strains from IBK and non-IBK eyes. The RTX operon was found at a reduced frequency in non-IBK eyes, though this frequency still exceeded 50% in non-IBK eyes within USMARC and UNL animal populations. This might be a useful metric to track in other cattle populations, especially those with frequent IBK outbreaks. In contrast, AR genes within a previously characterized genomic island were not detected at all in strains from non-IBK eyes. This island is associated with an increased AR phenotype [35]. The island did not always contain all ten AR genes but, some assortment of its AR genes, was found in 22 genotype 1 genomes (S1 Table). However, the 12% frequency of this island in genotype 1 strains may not accurately reflect the frequency of this type of resistance in IBK-associated *M*. *bovoculi* in the US. While the strains utilized in this paper from [12] were geographically biased heavily toward the US state of Nebraska, those with the island containing AR genes tended to be more frequently associated with out-of-state case submissions (right-tailed Fisher’s exact test p = 0.043, Odds Ratio = 2, CI_95%_ 1–6) (Table 1).

Due to high recombination in genotype 1, subtypes a-d are separated by very short branches and contain no tagging SNPs though their clade support was high. This was despite the removal of ~4K SNPs with the strongest recombination signal. As such, though there may be differences in pathogenicity potential among the subtypes, rampant recombination has obscured these differences at the SNP level. This highlights the difficulty of leveraging genomic data to immediate control solutions such as vaccines in the face of organisms with high recombination rates.

In three *M*. *bovoculi* strains, the *M*. *bovis* 16S mosaic import corresponds to the latter 80% of variable region 6 (V6) nucleotides [61] (Table 2). This is noteworthy since V6 has been found to be the most discriminatory of the nine 16S variable regions [62]. Mosaicism at this locus may not be uncommon to *Moraxella bovoculi* since we not only found evidence of it in 3 of 220 (1.4%) genotype 1 strains, but also found 3 rRNA sequences (JN001939, JN001941, and JN001949) featuring the same mosaic pattern in the GenBank nr database. These GenBank samples have been identified, we believe incorrectly, as *M*. *bovis*. Such sequence-based misidentifications are a foreseeable consequence of 16S V6 mosaicism in *M*. *bovoculi*. Alternative identifications methods, such as MALDI-TOF MS [37] may be more useful to correctly identify these strains, as this proteomic method would potentially be less impacted by single gene mosaicism. These methods have already demonstrated utility to discriminate *M*. *bovoculi* and *M*. *bovis*, including isolates with mosaicism, [37] with iterative improvements to the MALDI-TOF MS models and databases planned as new whole genome sequence-based identifications become available.

While sequence-based misidentification of *Moraxella* from IBK cases is a legitimate cause for concern, the import of large portions of virulence factor genes, such as RTX from *M*. *bovis* lends further subjective evidence that *M*. *bovoculi* genotype 1 strains have increased potential to have a primary role in IBK pathogenesis [25]. Four different segments of the RTX operon have been imported from *M*. *bovis* and ten *M*. *bovoculi* strains contain 1–2 of these segments (Table 2). These segments are quite large, comprising as much as 40% and 66% of *mbxB* and *mbxD* respectively. These two genes form a stable inner-membrane complex, that along with *tolC* create the channel for transporting hemolysins across the bacterial cell membrane [63]. Interspecies recombination may partially explain RTX-based vaccine failure in treating IBK [60] and may serve as a way for pathogenic *Moraxella sp*. to ‘experiment’ with different toxin transport configurations in the face of anthropogenic selection. The hemolysis and/or leukotoxicity phenotypes of such inter-species recombinant configurations should be investigated *in vitro*. These also might be important challenge models to test *in vivo*. Perhaps other *M*. *bovoculi* strains possess adequate pathogenesis factors to reproduce disease in experimental models though the type strain could not [3].

## Conclusion

US *M*. *bovoculi* from cattle eyes place into two major genotypes. Genotype 1 frequently contains the repeats-in-toxin putative virulence factor and, more rarely, a genomic island with up to ten antibiotic resistance genes. Genotype 1, to-date, is the only genotype identified from clinical infectious bovine keratoconjunctivitis cases. Due to very high recombination, subtypes of Genotype 1 cannot be distinguished at the SNP level, though these subtypes may vary in their potential for virulence. Interspecies recombination with *M*. *bovis* indicates that, for at least two-loci, these species share a common gene pool. These observations suggest that there are potentially complex roles of *M*. *bovoculi* in the etiology of IBK. Because of this, future IBK vaccine development may benefit from the identification of conserved outer membrane proteins shared by both *Moraxella* species.

## Supporting information

S1 Dataset *M. bovoculi* genotype 1 snp aligment minus recombinant snps. (ZIP)

S1 Table Collection and genomic information for *M. bovoculi* strains. (DOCX)

S2 Table SNPs diagnostic for *M. bovoculi* genotypes 1 and 2. (CSV)
