# Supplementary material for: Whole genome sequencing of Moraxella bovoculi reveals high genetic diversity and evidence for interspecies recombination at multiple loci
Source: PLoS One. 2018 Dec 17;13(12):e0209113. doi: 10.1371/journal.pone.0209113 (PMC6296526; doi:10.1371/journal.pone.0209113)
Supplement: S1 Table — (DOCX) [file pone.0209113.s002.docx]

**S1 Table: Collection and genomic element information for *M. bovoculi* strains**

| **Strain identifier** | **IBK Signs** | **Collection location** | **Additional information** | **Antibiotic resistance genes from island -the gene order in CP011374 is *bla*_ROB-1_, *sul2*, *strA*, *aph(3’)-Ic*, *tet(H)*, *msr(E)*, *mph(E)*, *sul2*, *floR*, *tet(H)*** | **RTX** | **Genotype** |
| --- | --- | --- | --- | --- | --- | --- |
| Mb57904 | + | California, USA |  | 1-5,8-10 | + | 1 |
| Mb57884 | + | Indiana, USA |  | 1-5,8,10 | + | 1 |
| Mb58065 | + | Indiana, USA |  | 1-5,8,10 | - | 1 |
| Mb57892 | + | Indiana, USA |  | 1-5,8-10 | + | 1 |
| Mb57923 | + | Minnesota, USA |  | 1-5,8,10 | - | 1 |
| Mb58063 | + | Minnesota, USA |  | 1-5,8,10 | + | 1 |
| Mb58080 | + | Minnesota, USA |  | 2,4-10 | + | 1 |
| Mb57916 | + | Nebraska, USA |  | 1-10 | + | 1 |
| Mb58069 | + | Nebraska, USA |  | 1-10 | + | 1 |
| Mb57871 | + | Nebraska, USA |  | 1-5,8,10 | + | 1 |
| Mb58119 | + | Nebraska, USA |  | 1-5,8-10 | + | 1 |
| Mb57864 | + | Nebraska, USA |  | 2,4-10 | + | 1 |
| Mb58121 | + | Nebraska, USA |  | 2,4-10 | + | 1 |
| Mb58122 | + | Nebraska, USA |  | 2-8,10 | - | 1 |
| Mb57881 | + | Nebraska, USA |  | 5,10 | - | 1 |
| Mb57899 | + | Nebraska, USA |  | 5,10 | + | 1 |
| Mb58101 | + | Ohio, USA |  | 1-5,8,10 | + | 1 |
| Mb57855 | + | Ohio, USA |  | 2,4-5,8-10 | + | 1 |
| Mb57857 | + | Texas, USA |  | 2,4-10 | + | 1 |
| Mb58027 | + | Virginia, USA |  | 1-10 | + | 1 |
| Mb57887 | + | Wisconsin, USA |  | 1-5,8,10 | + | 1 |
| Mb57891 | + | Wisconsin, USA |  | 2,4-10 | + | 1 |
| Mb57903 | + | California, USA |  |  | + | 1 |
| Mb58011 | + | Illinois, USA |  |  | + | 1 |
| Mb58015 | + | Illinois, USA |  |  | + | 1 |
| Mb58016 | + | Illinois, USA |  |  | + | 1 |
| Mb58035 | + | Illinois, USA |  |  | + | 1 |
| Mb58050 | + | Illinois, USA |  |  | + | 1 |
| Mb58090 | + | Illinois, USA |  |  | - | 1 |
| Mb57863 | + | Indiana, USA |  |  | + | 1 |
| Mb57860 | + | Iowa, USA |  |  | + | 1 |
| Mb57893 | + | Iowa, USA |  |  | + | 1 |
| Mb57905 | + | Iowa, USA |  |  | + | 1 |
| Mb58036 | + | Iowa, USA |  |  | - | 1 |
| Mb58123 | + | Iowa, USA |  |  | - | 1 |
| Mb57885 | + | Kansas, USA |  |  | + | 1 |
| Mb57894 | + | Kansas, USA |  |  | + | 1 |
| Mb57922 | + | Kansas, USA |  |  | + | 1 |
| Mb58017 | + | Kansas, USA |  |  | + | 1 |
| Mb58030 | + | Kansas, USA |  |  | + | 1 |
| Mb58039 | + | Kansas, USA |  |  | + | 1 |
| Mb58054 | + | Kansas, USA |  |  | - | 1 |
| Mb58055 | + | Kansas, USA |  |  | - | 1 |
| Mb58079 | + | Kansas, USA |  |  | - | 1 |
| Mb58081 | + | Minnesota, USA |  |  | + | 1 |
| Mb58088 | + | Minnesota, USA |  |  | + | 1 |
| Mb57851 | + | Montana, USA |  |  | + | 1 |
| Mb57906 | + | Montana, USA |  |  | + | 1 |
| Mb58000 | + | Montana, USA |  |  | + | 1 |
| Mb58001 | + | Montana, USA |  |  | + | 1 |
| Mb58009 | + | Montana, USA |  |  | + | 1 |
| Mb58040 | + | Montana, USA |  |  | + | 1 |
| Mb58053 | + | Montana, USA |  |  | + | 1 |
| Mb58082 | + | Montana, USA |  |  | + | 1 |
| Mb57850 | + | Nebraska, USA |  |  | + | 1 |
| Mb57852 | + | Nebraska, USA |  |  | + | 1 |
| Mb57853 | + | Nebraska, USA |  |  | + | 1 |
| Mb57854 | + | Nebraska, USA |  |  | + | 1 |
| Mb57862 | + | Nebraska, USA |  |  | + | 1 |
| Mb57865 | + | Nebraska, USA |  |  | + | 1 |
| Mb57866 | + | Nebraska, USA |  |  | + | 1 |
| Mb57867 | + | Nebraska, USA |  |  | + | 1 |
| Mb57869 | + | Nebraska, USA |  |  | + | 1 |
| Mb57870 | + | Nebraska, USA |  |  | + | 1 |
| Mb57872 | + | Nebraska, USA |  |  | + | 1 |
| Mb57873 | + | Nebraska, USA |  |  | + | 1 |
| Mb57874 | + | Nebraska, USA |  |  | + | 1 |
| Mb57875 | + | Nebraska, USA |  |  | + | 1 |
| Mb57876 | + | Nebraska, USA |  |  | - | 1 |
| Mb57877 | + | Nebraska, USA |  |  | + | 1 |
| Mb57878 | + | Nebraska, USA |  |  | + | 1 |
| Mb57880 | + | Nebraska, USA |  |  | + | 1 |
| Mb57882 | + | Nebraska, USA |  |  | + | 1 |
| Mb57883 | + | Nebraska, USA |  |  | - | 1 |
| Mb57888 | + | Nebraska, USA |  |  | + | 1 |
| Mb57889 | + | Nebraska, USA |  |  | + | 1 |
| Mb57895 | + | Nebraska, USA |  |  | + | 1 |
| Mb57896 | + | Nebraska, USA |  |  | + | 1 |
| Mb57897 | + | Nebraska, USA |  |  | + | 1 |
| Mb57898 | + | Nebraska, USA |  |  | + | 1 |
| Mb57900 | + | Nebraska, USA |  |  | + | 1 |
| Mb57901 | + | Nebraska, USA |  |  | + | 1 |
| Mb57902 | + | Nebraska, USA |  |  | + | 1 |
| Mb57907 | + | Nebraska, USA |  |  | + | 1 |
| Mb57908 | + | Nebraska, USA |  |  | + | 1 |
| Mb57909 | + | Nebraska, USA |  |  | - | 1 |
| Mb57910 | + | Nebraska, USA |  |  | + | 1 |
| Mb57911 | + | Nebraska, USA |  |  | + | 1 |
| Mb57912 | + | Nebraska, USA |  |  | + | 1 |
| Mb57913 | + | Nebraska, USA |  |  | + | 1 |
| Mb57914 | + | Nebraska, USA |  |  | + | 1 |
| Mb57915 | + | Nebraska, USA |  |  | + | 1 |
| Mb57917 | + | Nebraska, USA |  |  | + | 1 |
| Mb57921 | + | Nebraska, USA |  |  | + | 1 |
| Mb57924 | + | Nebraska, USA |  |  | + | 1 |
| Mb57925 | + | Nebraska, USA |  |  | + | 1 |
| Mb57967 | + | Nebraska, USA |  |  | + | 1 |
| Mb57993 | + | Nebraska, USA |  |  | + | 1 |
| Mb57999 | + | Nebraska, USA |  |  | + | 1 |
| Mb58002 | + | Nebraska, USA |  |  | + | 1 |
| Mb58003 | + | Nebraska, USA |  |  | + | 1 |
| Mb58004 | + | Nebraska, USA |  |  | + | 1 |
| Mb58005 | + | Nebraska, USA |  |  | + | 1 |
| Mb58006 | + | Nebraska, USA |  |  | + | 1 |
| Mb58007 | + | Nebraska, USA |  |  | + | 1 |
| Mb58008 | + | Nebraska, USA |  |  | + | 1 |
| Mb58012 | + | Nebraska, USA |  |  | + | 1 |
| Mb58013 | + | Nebraska, USA |  |  | + | 1 |
| Mb58014 | + | Nebraska, USA |  |  | + | 1 |
| Mb58018 | + | Nebraska, USA |  |  | + | 1 |
| Mb58019 | + | Nebraska, USA |  |  | + | 1 |
| Mb58020 | + | Nebraska, USA |  |  | + | 1 |
| Mb58022 | + | Nebraska, USA |  |  | + | 1 |
| Mb58023 | + | Nebraska, USA |  |  | + | 1 |
| Mb58024 | + | Nebraska, USA |  |  | + | 1 |
| Mb58025 | + | Nebraska, USA |  |  | + | 1 |
| Mb58031 | + | Nebraska, USA |  |  | + | 1 |
| Mb58032 | + | Nebraska, USA |  |  | + | 1 |
| Mb58033 | + | Nebraska, USA |  |  | + | 1 |
| Mb58034 | + | Nebraska, USA |  |  | - | 1 |
| Mb58037 | + | Nebraska, USA |  |  | - | 1 |
| Mb58038 | + | Nebraska, USA |  |  | + | 1 |
| Mb58041 | + | Nebraska, USA |  |  | + | 1 |
| Mb58049 | + | Nebraska, USA |  |  | + | 1 |
| Mb58051 | + | Nebraska, USA |  |  | + | 1 |
| Mb58052 | + | Nebraska, USA |  |  | + | 1 |
| Mb58056 | + | Nebraska, USA |  |  | + | 1 |
| Mb58057 | + | Nebraska, USA |  |  | + | 1 |
| Mb58058 | + | Nebraska, USA |  |  | - | 1 |
| Mb58060 | + | Nebraska, USA |  |  | + | 1 |
| Mb58061 | + | Nebraska, USA |  |  | + | 1 |
| Mb58062 | + | Nebraska, USA |  |  | + | 1 |
| Mb58064 | + | Nebraska, USA |  |  | + | 1 |
| Mb58066 | + | Nebraska, USA |  |  | + | 1 |
| Mb58067 | + | Nebraska, USA |  |  | - | 1 |
| Mb58068 | + | Nebraska, USA |  |  | + | 1 |
| Mb58070 | + | Nebraska, USA |  |  | + | 1 |
| Mb58071 | + | Nebraska, USA |  |  | + | 1 |
| Mb58072 | + | Nebraska, USA |  |  | + | 1 |
| Mb58073 | + | Nebraska, USA |  |  | + | 1 |
| Mb58074 | + | Nebraska, USA |  |  | + | 1 |
| Mb58075 | + | Nebraska, USA |  |  | - | 1 |
| Mb58076 | + | Nebraska, USA |  |  | + | 1 |
| Mb58083 | + | Nebraska, USA |  |  | + | 1 |
| Mb58084 | + | Nebraska, USA |  |  | + | 1 |
| Mb58085 | + | Nebraska, USA |  |  | + | 1 |
| Mb58091 | + | Nebraska, USA |  |  | - | 1 |
| Mb58092 | + | Nebraska, USA |  |  | + | 1 |
| Mb58093 | + | Nebraska, USA |  |  | + | 1 |
| Mb58094 | + | Nebraska, USA |  |  | - | 1 |
| Mb58095 | + | Nebraska, USA |  |  | + | 1 |
| Mb58096 | + | Nebraska, USA |  |  | + | 1 |
| Mb58102 | + | Nebraska, USA |  |  | + | 1 |
| Mb58108 | + | Nebraska, USA |  |  | - | 1 |
| Mb58118 | + | Nebraska, USA |  |  | + | 1 |
| Mb60475 | - | Nebraska, USA | Age: ~3 months, University of Nebraska, Lincoln ARDC herd |  | + | 1 |
| Mb60476 | - | Nebraska, USA | Age: ~3 months, University of Nebraska, Lincoln ARDC herd |  | - | 1 |
| Mb60477 | - | Nebraska, USA | Age: ~3 months, University of Nebraska, Lincoln ARDC herd |  | + | 1 |
| Mb60478 | - | Nebraska, USA | Age: ~3 months, University of Nebraska, Lincoln ARDC herd |  | - | 1 |
| Mb60479 | - | Nebraska, USA | Age: 4 years, University of Nebraska, Lincoln ARDC herd |  | - | 1 |
| Mb60481 | - | Nebraska, USA | Deceased, Age: 9 days, Eyes normal- No diagnosis |  | - | 1 |
| Mb68485 | - | Nebraska, USA | US Meat Animal Research Center |  | - | 1 |
| Mb68486 | - | Nebraska, USA | US Meat Animal Research Center |  | - | 1 |
| Mb68487 | - | Nebraska, USA | US Meat Animal Research Center |  | + | 1 |
| Mb68488 | - | Nebraska, USA | US Meat Animal Research Center |  | + | 1 |
| Mb68489 | - | Nebraska, USA | US Meat Animal Research Center |  | + | 1 |
| Mb68490 | - | Nebraska, USA | US Meat Animal Research Center |  | + | 1 |
| Mb68492 | - | Nebraska, USA | US Meat Animal Research Center |  | + | 1 |
| Mb68496 | - | Nebraska, USA | US Meat Animal Research Center |  | + | 1 |
| Mb68506 | - | Nebraska, USA | US Meat Animal Research Center |  | + | 1 |
| Mb68507 | - | Nebraska, USA | US Meat Animal Research Center |  | - | 1 |
| Mb68508 | - | Nebraska, USA | US Meat Animal Research Center |  | + | 1 |
| Mb68510 | - | Nebraska, USA | US Meat Animal Research Center |  | + | 1 |
| Mb68511 | - | Nebraska, USA | US Meat Animal Research Center |  | - | 1 |
| Mb68512 | - | Nebraska, USA | US Meat Animal Research Center |  | - | 1 |
| Mb68513 | - | Nebraska, USA | US Meat Animal Research Center |  | - | 1 |
| Mb68514 | - | Nebraska, USA | US Meat Animal Research Center |  | + | 1 |
| Mb68517 | - | Nebraska, USA | US Meat Animal Research Center |  | + | 1 |
| Mb68519 | - | Nebraska, USA | US Meat Animal Research Center |  | + | 1 |
| Mb68522 | - | Nebraska, USA | US Meat Animal Research Center |  | + | 1 |
| Mb68527 | - | Nebraska, USA | US Meat Animal Research Center |  | + | 1 |
| Mb68528 | - | Nebraska, USA | US Meat Animal Research Center |  | - | 1 |
| Mb68529 | - | Nebraska, USA | US Meat Animal Research Center |  | - | 1 |
| Mb68531 | - | Nebraska, USA | US Meat Animal Research Center |  | + | 1 |
| Mb68532 | - | Nebraska, USA | US Meat Animal Research Center |  | + | 1 |
| Mb68536 | - | Nebraska, USA | US Meat Animal Research Center |  | + | 1 |
| Mb68541 | - | Nebraska, USA | US Meat Animal Research Center |  | - | 1 |
| Mb68542 | - | Nebraska, USA | US Meat Animal Research Center |  | - | 1 |
| Mb68551 | - | Nebraska, USA | US Meat Animal Research Center |  | + | 1 |
| Mb68552 | - | Nebraska, USA | US Meat Animal Research Center |  | - | 1 |
| Mb68554 | - | Nebraska, USA | US Meat Animal Research Center |  | - | 1 |
| Mb68555 | - | Nebraska, USA | US Meat Animal Research Center |  | - | 1 |
| Mb68495 | - | Nebraska, USA | US Meat Animal Research Center |  |  | 2 |
| Mb68499 | - | Nebraska, USA | US Meat Animal Research Center |  |  | 2 |
| Mb68500 | - | Nebraska, USA | US Meat Animal Research Center |  |  | 2 |
| Mb68501 | - | Nebraska, USA | US Meat Animal Research Center |  |  | 2 |
| Mb68502 | - | Nebraska, USA | US Meat Animal Research Center |  |  | 2 |
| Mb68503 | - | Nebraska, USA | US Meat Animal Research Center |  |  | 2 |
| Mb68504 | - | Nebraska, USA | US Meat Animal Research Center |  |  | 2 |
| Mb68505 | - | Nebraska, USA | US Meat Animal Research Center |  |  | 2 |
| Mb68515 | - | Nebraska, USA | US Meat Animal Research Center |  |  | 2 |
| Mb68516 | - | Nebraska, USA | US Meat Animal Research Center |  |  | 2 |
| Mb68520 | - | Nebraska, USA | US Meat Animal Research Center |  |  | 2 |
| Mb68523 | - | Nebraska, USA | US Meat Animal Research Center |  |  | 2 |
| Mb68524 | - | Nebraska, USA | US Meat Animal Research Center |  |  | 2 |
| Mb68525 | - | Nebraska, USA | US Meat Animal Research Center |  |  | 2 |
| Mb68533 | - | Nebraska, USA | US Meat Animal Research Center |  |  | 2 |
| Mb68534 | - | Nebraska, USA | US Meat Animal Research Center |  |  | 2 |
| Mb68535 | - | Nebraska, USA | US Meat Animal Research Center |  |  | 2 |
| Mb68538 | - | Nebraska, USA | US Meat Animal Research Center |  |  | 2 |
| Mb68539 | - | Nebraska, USA | US Meat Animal Research Center |  |  | 2 |
| Mb68540 | - | Nebraska, USA | US Meat Animal Research Center |  |  | 2 |
| Mb68543 | - | Nebraska, USA | US Meat Animal Research Center |  |  | 2 |
| Mb68544 | - | Nebraska, USA | US Meat Animal Research Center |  |  | 2 |
| Mb68545 | - | Nebraska, USA | US Meat Animal Research Center |  |  | 2 |
| Mb68546 | - | Nebraska, USA | US Meat Animal Research Center |  |  | 2 |
| Mb68547 | - | Nebraska, USA | US Meat Animal Research Center |  |  | 2 |
| Mb68549 | - | Nebraska, USA | US Meat Animal Research Center |  |  | 2 |
| Mb58042 | + | New York, USA |  |  | + | 1 |
| Mb57918 | + | North Dakota, USA |  |  | + | 1 |
| Mb57879 | + | Oklahoma, USA |  |  | + | 1 |
| Mb58026 | + | Oklahoma, USA |  |  | - | 1 |
| Mb58043 | + | Oklahoma, USA |  |  | + | 1 |
| Mb58044 | + | Oklahoma, USA |  |  | - | 1 |
| Mb58097 | + | South Carolina, USA |  |  | + | 1 |
| Mb57861 | + | South Dakota, USA |  |  | - | 1 |
| Mb58028 | + | South Dakota, USA |  |  | - | 1 |
| Mb58078 | + | South Dakota, USA |  |  | + | 1 |
| Mb58107 | + | South Dakota, USA |  |  | + | 1 |
| Mb58010 | + | Tennessee, USA |  |  | + | 1 |
| Mb57992 | + | Texas, USA |  |  | + | 1 |
| Mb58046 | + | Texas, USA |  |  | + | 1 |
| Mb57858 | + | Vermont, USA |  |  | + | 1 |
| Mb57890 | + | Vermont, USA |  |  | + | 1 |
| Mb58047 | + | Virginia, USA |  |  | - | 1 |
| Mb58048 | + | Virginia, USA |  |  | + | 1 |
| Mb58059 | + | Virginia, USA |  |  | + | 1 |
| Mb58086 | + | Virginia, USA |  |  | - | 1 |
| Mb58103 | + | Virginia, USA |  |  | + | 1 |
| Mb58111 | + | Virginia, USA |  |  | + | 1 |
| Mb57919 | + | Washington, USA |  |  | + | 1 |
| Mb58087 | + | West Virginia, USA |  |  | + | 1 |
| Mb57886 | + | Wisconsin, USA |  |  | + | 1 |
| Mb58029 | + | Wisconsin, USA |  |  | - | 1 |
| Mb57920 | + | Wyoming, USA |  |  | + | 1 |
| Mb58099 | + | Wyoming, USA |  |  | + | 1 |
